# Compost Tea as a Natural Bioactive Solution: Unlocking the Antimicrobial and Antiviral Potential of Bell Pepper and Citrus Wastes

**DOI:** 10.1111/1758-2229.70260

**Published:** 2026-01-27

**Authors:** Maria Vittoria Verrillo, Roberta Della Marca, Vincenza Cozzolino, Annalisa Chianese, Carla Zannella, Massimiliano Galdiero, Riccardo Spaccini, Anna De Filippis

**Affiliations:** ^1^ Department of Agricultural Sciences University of Naples Federico II Naples Italy; ^2^ Interdepartmental Research Centre on Nuclear Magnetic Resonance (NMR) for the Environment, Agro‐Food and New Materials (CERMANU), University of Naples Federico II Naples Italy; ^3^ Department of Experimental Medicine University of Campania “L.Vanvitelli” Naples Italy; ^4^ Complex Operative Unit of Virology and Microbiology, University Hospital of Campania “Luigi Vanvitelli” Naples Italy

## Abstract

The growing demand for sustainable medical applications has sparked interest in the valorisation of agro‐industrial waste for bioactive compounds. Compost teas (CTs) from agrifood waste, rich in phenolics and lignin derivatives, offer promising biological properties. This study analysed CTs from bell pepper (CT‐BP) and citrus (CT‐C) composted waste, assessing their antioxidant, antiviral, and antimicrobial activities. NMR spectroscopy and thermochemolysis revealed that CT‐BP had more oxidised lignin derivatives, while CT‐C contained intact lignin structures. Both CTs effectively inhibited Gram‐positive bacteria ( *Staphylococcus aureus* and *Enterococcus faecalis* ), with CT‐BP showing greater efficacy. However, Gram‐negative bacteria ( *Escherichia coli* and *Pseudomonas aeruginosa* ) were more resistant. CT‐BP also exhibited potent antiviral effects against enveloped viruses like herpes simplex virus type 1 (HSV‐1) and respiratory syncytial virus (RSV). These findings support the use of compost‐derived extracts as sustainable bioactive agents, offering natural alternatives to conventional treatments. Further research could enhance the extraction and scalability of these materials for biomedical applications, aligning with principles of the circular economy.

## Graphical abstract

Compost teas derived from bell pepper and citrus waste are rich in bioactive phenolics and lignin derivatives, showing strong antibacterial activity against gram‐positive bacteria and potent antiviral effects against HSV‐1 and RSV. These findings highlight their potential as sustainable, circular bioresources for biomedical applications.

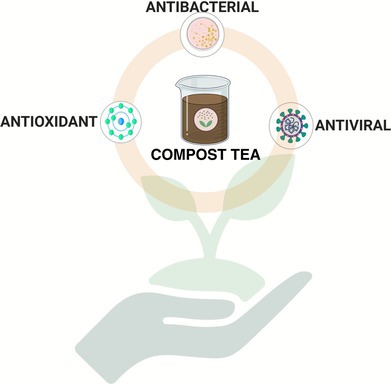

## 1 Introduction

The need for innovative, sustainable, and effective antimicrobial strategies has become increasingly urgent due to the rising threat of viral and bacterial outbreaks, as well as the limitations in discovering new drugs (Sharma et al. 2023). Bio‐based solutions derived from organic waste offer a compelling approach to addressing these challenges. This approach aligns with the principles of the circular economy by repurposing waste into valuable resources, thereby contributing to both health and environmental sustainability (Wagh et al. 2024). The isolation of active biochemical intermediates and products from organic waste, such as agricultural residues, is a valuable strategy for minimising environmental impacts while harnessing the bioactive potential of natural products. Among these, compost tea (CT)—a liquid extract produced through the fermentation of composted organic matter—has emerged as a promising candidate. Traditionally recognised for its applications in agriculture to suppress plant pathogens and promote crop health and resilience, CT also shows promise for broader applications, including the prevention of microbial infections (Marin et al. 2013; Verrillo, Cozzolino, et al. 2021; Verrillo, Salzano, et al. 2021). Its complex composition, including bioactive compounds, micronutrients, and diverse microbial consortia, provides a unique opportunity for exploring its antimicrobial activity against human pathogens. Globally, the increasing generation of agrifood waste from citrus and bell peppers presents a chance to transform these byproducts into compost teas rich in bioactive metabolites. Citrus wastes, such as peels and pulp, are known to retain flavonoids, limonoids, and essential oils with documented antimicrobial and antiviral properties. For instance, flavonones like hesperidin and naringenin have demonstrated inhibitory effects against several human viruses, including influenza and coronaviruses (Saini et al. 2022). Similarly, bell pepper waste, rich in capsaicinoids and phenolic compounds, has been shown to exhibit antimicrobial and antiviral activities, likely through mechanisms involving disruption of bacterial and viral membranes or inhibition of microbial replication (Periferakis et al. 2023). In addition, during the composting process, these bioactive molecules are transformed, and beneficial microbial communities flourish, further enhancing the potential of the resulting CT to interfere with microbial infections (Razola‐Diaz et al. 2022). The fermentation phase in compost tea preparation facilitates the release of cellular synthetases into a bioavailable liquid form while supporting the additional development of microbial metabolites with potential synergistic antimicrobial effects. Moreover, CT is increasingly recognised for its multifaceted biological activities, including the antioxidant ones (Seth et al. 2023) due to its high content of phenols, flavonoids, and other redox‐mediating agents. Studies have shown that CT made from nutrient‐rich wastes such as citrus peels and vegetable scraps exhibits significant antioxidant capacity, comparable to synthetic antioxidants (Siddique et al. 2024). The antimicrobial activity of compost tea stems from its complex microbiota, comprising bacteria, fungi, and actinomycetes, which produce secondary metabolites that inhibit a wide range of pathogens, including bacteria, fungi, and some protozoa (Verrillo, Cozzolino, et al. 2021; Verrillo, Salzano, et al. 2021). This study aims to evaluate the antiviral efficacy of compost teas extracted from bell pepper (CT‐BP), citrus (CT‐C), and agrifood waste against selected human viruses. The goal is to establish a scientific basis for the application of compost teas in human health, highlighting their potential to inhibit viral replication, reduce infectivity, and enhance host immunity. The findings may inform the development of innovative, eco‐friendly solutions that support circular economy principles and promote sustainable health practices.

## 2 Material and Methods

### 2.1 Composting Process and Extraction of Compost Tea

The composting of agrifood residues was carried out at the Experimental Farm of the University of Napoli Federico II (Dept. of Agricultural Sciences). Vegetable residues included horticultural by‐products from bell pepper cultivation and industrial lemon‐processing waste. Each biomass was mixed with maize straw and poplar wood chips as bulking agents (70:30 w/w) to form a balanced composting mixture. The homogenised materials were arranged into static piles over perforated rubber tubing, with airflow provided by a rotary pump to maintain aerobic conditions. The composting process lasted 100 days, including a thermophilic phase at 55°C–65°C and a mesophilic phase at 30°C–40°C, followed by a curing stage (Verrillo, Cozzolino, et al. 2021; Verrillo, Salzano, et al. 2021). After maturation, 1 kg samples were randomly collected from each pile, air‐dried, ground, and sieved (2 mm), and then stored at 4°C for further analysis. To prepare compost tea (CT), 200 g of compost was placed in gauze bags and submerged in 1 L of distilled water (1:5 w/v). Air was actively insufflated for 5 min every 3 h using an automatic aeration system. After 7 days of aeration, the resulting solution was freeze‐dried and stored at 4°C for subsequent analyses and experimental use.

### 2.2 NMR Spectroscopy

The solid‐state ^13^C CPMAS NMR spectra of the compost tea (CT) samples were acquired on a Bruker AV‐300 spectrometer, fitted with a 4 mm wide‐bore MAS probe. Cross‐polarisation was achieved with composite ‘ramp’ pulse complemented by preliminarily optimised experimental parameters: rotor spin rate of 13,000 Hz, recycle time of 2 s, 92 W of 1H‐power for cross‐polarisation (CP), a 1H 90° pulse duration of 2.85 μs, 150 W of ^13^C power for CP, a 1 ms contact time, and a 30 ms acquisition time with 4000 scans. The samples were packed into 4 mm zirconium rotors with Kel‐F caps. The free induction decay (FID) was processed by applying a 4 k zero filling and an exponential filter function with 150 Hz of line broadening. The chemical shift range was divided into six regions corresponding to major organic functional groups: 0–45 ppm (aliphatic‐C), 45–60 ppm (methoxyl‐C and N‐alkyl‐C), 60–110 ppm (O‐alkyl‐C), 110–145 ppm (aromatic‐C), 145–160 ppm (O‐aryl‐C), and 160–190 ppm (carboxyl‐C). The percentage contribution of each carbon group was calculated by dividing the area of the corresponding spectral interval (Riabs) by the sum of all spectral interval areas, as follows: Ri%=∑RiabsRiabs×100.

The structural properties of compost extracts may be inferred by the calculation of dimensionless indexes issued from the combination of specific spectral areas (Verrillo et al. 2023; Altieri et al. 2024): O‐Alkyl ratio (A/OA = [(0–45)/(60–110)]), hydrophobic index (HB = ∑[(0–45) + (45–60)/2 + (110–160)]/∑[(45–60)/2 + (60–110) + (160–190)]), and lignin ratio (LigR = [(45–60)/(140–160)]). These indices are used to assess the biochemical stability and bioactive properties of compost extracts (Verrillo, Cozzolino, et al. 2021; Verrillo, Salzano, et al. 2021).

### 2.3 Offline Pyrolysis Gas Chromatography–Mass Spectrometry (TMAH‐GC–MS)

For the offline thermally assisted hydrolysis and methylation (TMAH) analysis, 500 mg of dried compost tea (CT) was mixed with 1 mL of a 25% TMAH solution in methanol. and placed in a Pyrex tubular reactor (50 cm × 3.5 cm i.d.) heated to 400°C for 30 min in a round furnace (Barnstead Thermolyne).

The untied monomers released during the thermochemolysis were continuously swept by a helium flow (20 mL/min) into two chloroform traps (50 mL each) maintained in an ice/salt bath. The collected solutions were combined and evaporated under reduced pressure using a rotary evaporator. The remaining residue was dissolved in 1 mL of chloroform and transferred to a glass vial for subsequent GC–MS analysis. The GC–MS was performed on a Perkin‐Elmer Autosystem XL, equipped with an RTX‐5MS WCOT capillary column (Restek, 30 m × 0.25 mm; 0.25 μm film thickness). A heated transfer line (250°C) connected the column to a PE Turbomass‐Gold quadrupole mass spectrometer. The chromatographic separation was run on the following program set up: an initial isothermal step at 60°C for 1 min, followed by a 7°C/min ramp to 320°C, held isothermally for 10 min. Helium served as the carrier gas at a flow rate of 1.90 mL/min, the injector temperature was set to 250°C, and a 30 mL/min split flow was used. Mass spectra were recorded in electron impact (EI) mode at 70 eV, scanning the range 45–650 m/z with a cycle time of 1 s. Offline pyrolysis gas chromatography–mass spectrometry (TMAH‐GC–MS) was performed, and the resulting spectra were compared with mass spectral libraries, including NIST and Wiley databases, to identify the extracted compounds. Before analysis, samples were derivatized using tetramethylammonium hydroxide (TMAH) to allow the detection and quantification of polar compounds (Zang et al. 2001; Kuroda et al. 2002; Geffroy‐Rodier et al. 2009).

### 2.4 Antioxidant Performance and Total Phenolic Content

The antioxidant activity of compost tea was assessed using both the ABTS and DPPH assays. (Verrillo et al. 2025). The ABTS technique relies on the oxidation of 2,2′‐azinobis‐(3‐ethylbenzothiazoline‐6‐sulfonic acid) diammonium salt (ABTS) by potassium persulfate, resulting in the formation of the ABTS radical cation (ABTS•+). In brief, a 7 mM ABTS stock solution was prepared, and the ABTS•+ was generated by adding 2.45 mM potassium persulfate, followed by incubation of the mixture in the dark at room temperature for 16 h. To prepare the working solution, 10 mL of the stock solution was diluted in 800 mL of a 50:50 (v/v) water/ethanol mixture, and its final absorbance at 734 nm was adjusted to 0.75–0.80 using a spectrophotometer. For the sample preparation, 4 mg of CT extract was dissolved in 2 mL of water, and 100 μL of this solution was added to 1.9 mL of the ABTS•+ working solution. After shaking for 2 min in the dark to allow for interaction between CT and the ABTS•+ radicals, the absorbance was measured at 734 nm using a PerkinElmer Lambda 25 UV/Vis Spectrometer. The DPPH (2,2‐diphenyl‐1‐picrylhydrazyl) assay was performed to evaluate the free radical scavenging activity. Briefly, 50 μL of a CT solution (1 mg/mL) was mixed with 150 μL of a 0.3 mM DPPH solution in ethanol. A blank was prepared by substituting 50 μL of Milli‐Q water for the CT solution. The mixture was vortexed for 2 min and incubated in the dark at room temperature for 20 min. The absorbance at 517 nm was measured.

### 2.5 Antimicrobial Activity

Antibacterial activity assays were carried out by diffusion disk assay (DDK) and broth microdilution method (MIC). Antimicrobial screening of CTs was performed against different bacterial microorganisms, all of which were procured from the American Type Culture Collection (ATCC), based in Manassas, Virginia, USA. Particularly, we carried out two gram‐positive bacterial strains, that is, *Staphylococcus aureus* ( *S. aureus* ) *ATCC 6538P and Enterococcus faecalis* ( *E. faecalis* ) *ATCC 29212*, and two gram‐negative bacteria, such as *Pseudomonas aeruginosa* ( *P. aeruginosa* ) *ATCC 27355* and *Escherichia coli* ( *E. coli* ) *ATCC 35218*. The DDK method, described by the National Committee for Clinical Laboratory Standards (NCCLS), involves measuring the diffusion of each material in agar plates inoculated with the bacterial strains. If the bacterial cells are susceptible to the substance being tested, a clear inhibition zone forms between the disc and the growing bacteria. Each CTs were diluted at a concentration of 2 mg mL^−1^ for testing. Each bacterial strain was cultured on nutrient agar plates and incubated at 37°C for 24 h. The bacterial inoculum was standardised by transferring colonies from the agar to a sterile saline solution to achieve a concentration of 10^8^ CFU/mL (0.5 McFarland), which corresponds to 50% transmittance at 580 nm (Coleman model 6120, Maywood, IL). A volume of 200 μL of each bacterial suspension was spread over Mueller‐Hinton agar plates, and 6.0 mm sterile discs were impregnated with 20 μL of each humic material. The plates were incubated at 37°C for 24 h. A mixture of Ampicillin and clavulanic acid at the same concentration of CTs tested was used as a control. The diameter of the inhibition zones was measured, and the experiment was conducted in triplicate. Antimicrobial activity was estimated as the percentage inhibition of microbial growth using the following formula:

$$\begin{array}{c} \text{Inhibition}\;\left(\%\right)=\text{Diameter of control Diameter of inhibition zone}\;\left(\text{sample}\right)\\ \;-\text{Diameter of control}\times 100. \end{array}$$

This approach allows a clear quantification of the inhibitory effect of compost tea on the tested microorganisms.

To validate the antimicrobial results, other tests have been performed to determine the MIC concentration. The broth microdilution method was conducted in Mueller–Hinton Broth using sterile 96‐well polypropylene microtiter plates. Serial twofold dilutions of each CT were prepared in the test wells, resulting in concentrations ranging from 10 to 1000 μg mL^−1^. Bacterial cells, grown overnight, were inoculated into the wells at a final concentration of approximately 5 × 10^5^ CFU/mL. The plates were incubated at 37°C for 24 h, and MIC values were determined by measuring the absorbance at 570 nm. The lowest concentration that showed no visible turbidity was recorded as the MIC.

### 2.6 Antiviral Efficacy

#### 2.6.1 Plaque Reduction Assays

The antiviral potential of CTs was investigated through plaque‐reduction assays, as previously reported (Zannella et al. 2023).

*Cercopithecus aethiops* kidney cells (Vero‐76, ATCC CRL 1587, Manassas, Virginia, United States) were purchased from the American Type Culture Collection (ATCC, Manassas, Virginia, United States). Cells were cultured in Dulbecco's Modified Eagle Medium (DMEM, Microtech, Naples, Italy) with 4.5 g/L of glucose (Microtech, Naples, Italy), 2 mM of L‐glutamine (Microtech, Naples, Italy), 100 IU/mL of penicillin–streptomycin solution (Himedia, Naples, Italy), and supplemented with 10% fetal bovine serum (FBS, Microgem, Naples, Italy) in a humidified atmosphere with 5% CO_2_ at 37°C. The viruses used were Herpes Simplex Virus type 1 (HSV‐1) strain SC16, Respiratory Syncytial virus (RSV) (ATCC VR‐1540, Manassas, Virginia, United States), Poliovirus Type 1 (PV‐1) strain Chat (ATCC VR‐1562, Manassas, Virginia, United States), propagated on Vero‐76 cells (Cometa et al. 2022).

In each antiviral assay (Zannella et al. 2023), Vero‐76 cells were seeded in 24‐well plates (1.2 × 10^5^ cells/well) and grown for 24 h at 37°C in 5% CO_2_. CTs were added to the medium without FBS at concentrations ranging from 0.195 to 200 μg/mL, and all viruses were used at a multiplicity of infection (MOI) of 0.01.

- Co‐treatment assay: CTs and virus were simultaneously incubated on Vero‐76 cells for 1 h at 37°C.
- Cell pre‐treatment assay: first, CTs were added to Vero‐76 cells for 1 h. Then, the compounds were removed, and cells were infected with viral suspensions for 1 h at 37°C.
- Virus pre‐treatment assay: CTs and virus were incubated for 1 h at 37°C at an MOI of 0.1. After that, each mixture (CTs + virus) was diluted so that the compound reached an inactive concentration and the virus was at a final MOI of 0.01. Dilutions were added to the Vero‐76 monolayer for 1 h at 37°C and 5% CO_2_.
- Post‐treatment assay: cells were previously infected with the viral suspension in DMEM w/o FBS for 1 h at 37°C. Then, the cell monolayer was washed to remove extracellular virions and treated with CTs for 1 h at 37°C.

After each of the listed treatments, the monolayer was washed, covered with the complete medium (10% FBS) supplemented with 3% carboxymethylcellulose (CMC) (Sigma, C5678, C5013), and incubated for 24 h or 48 h at 37°C and 5% CO_2_. Finally, cells were fixed with 4% formaldehyde (Sigma–Aldrich, St. Louis, MO, USA) and stained with crystal violet solution (0.5%). The infected cells without CTs represented the negative control (ctr−), while several reagents were used as positive controls (ctr+). In detail, Rhamnolipid M15RL (50 μg/mL) was used in the co‐treatment and virus pre‐treatment (Giugliano et al. 2021), dextran‐sulfate (1 μM) in cell pre‐treatment, and acyclovir (5 μM) in post‐treatment for HSV‐1 (Chianese et al. 2023); pleconaril (2 μg/mL) was used in co‐treatment and virus pre‐treatment, and WIN51711 (5 μg/mL) and protein 2C (10 μM) in cell pre‐treatment and post‐treatment, respectively, for PV‐1 (Ambrosino et al. 2023). Finally, for RSV, Hylin‐a1 (50 μM) was used in co‐treatment and virus pre‐treatment assays (Chianese et al. 2023).

The inhibition rate of viral infectivity was calculated by comparing the number of plaques counted in cells treated with CTs to the plaques detected in the ctr−, according to the following formula

$$\begin{array}{c} \%\text{𝑉𝑖𝑟𝑎𝑙 𝑖𝑛ℎ𝑖𝑏𝑖𝑡𝑖𝑜𝑛}=100\\ -\left[100\times \text{(𝑝𝑙𝑎𝑞𝑢𝑒𝑠 𝑐𝑜𝑢𝑛𝑡𝑒𝑑 𝑖𝑛 𝑡ℎ𝑒 𝑡𝑒𝑠𝑡 𝑠𝑎𝑚𝑝𝑙𝑒/𝑝𝑙𝑎𝑞𝑢𝑒𝑠 𝑐𝑜𝑢𝑛𝑡𝑒𝑑 𝑖𝑛 𝑡ℎ𝑒}\;\mathrm{ctr}-\right)]. \end{array}$$

The 50% inhibitory concentration (IC_50_) was calculated via linear regression analysis.

#### 2.6.2 Statistical Analysis

Each antiviral test was performed in triplicate and expressed as mean ± standard deviation (SD). One‐way ANOVA was followed by Dunnett's multiple comparisons test, and all the graphs were generated by GraphPad Prism (version 8.0.1; Software for 2D graphing and statistics; GraphPad Software Inc.: San Diego, CA, USA, 2018). The significance of the difference between treated and ctr− samples was obtained with Dunnett's test and ANOVA analysis. The *p*‐value < 0.05 was considered significant. Specific lower *p*‐values (e.g., *p* < 0.0001) were reported when applicable to indicate better the strength of the difference observed in the antiviral experiments.

## 3 Results and Discussion

### 3.1 Biomolecular Characterisation of Compost Teas

#### 3.1.1 NMR Spectroscopy

The ^13^C‐CPMAS NMR spectra of CT samples (Figure S1) showed that dissolved organic carbon is distributed among various functional groups. O‐alkyl‐C (60–110 ppm) and aryl‐C signals (110–160 ppm), associated with lignin‐cellulose components from agro‐food residues, accounted for 56%–62% of the relative area (Table 1). In both spectra, broad bands in the Alkyl‐C region (0–45 ppm) appeared as multiple resonances (Figure S1). These indicate the presence of methylene and methyl groups in linear, branched, short‐chain, or cyclic alkyl components. Examples include carboxylic acids, sterols, amino acids, and oligopeptides (Verrillo et al. 2023).

**TABLE 1:** Relative C distribution (%) over chemical shift regions (ppm) and index ratios^a^ derived from ^13^C‐CPMAS‐NMR spectra of compost teas.

	0–45	45–60	60–110	110–145	145–160	160–190				
	Alkyl‐C	Methoxyl‐C	O‐Alkyl‐C	Aromatic‐C	Phenol‐C	Carboxyl‐C	HB/HI	A/AO	ARM	LR
CT‐BP	24.9	14.1	21.7	23.7	5.3	10.3	1.2	1.1	0.4	2.7
CT‐C	18.7	13.9	30.7	16.9	4.9	14.9	0.7	0.6	0.3	2.8

^a^ HB (hydrophobic index) = Ʃ[(0–45) + (45–60)/2 + (110–160)]/Ʃ[(45–60)/2 + (60–110) + (160–190)]; A/AO (alkyl ratio) = (0–45)/(60–110); LR (lignin ratio) = (45–60)/(145–160)7.

The prominent peak, marked at 56 ppm, subtends a substantial amount of methoxyl side units in lignin molecules, with a possible contribution from C–N bonds of peptidic moieties. In the O‐alkyl‐C region (60–110 ppm), the signals at 72 ppm derive from the coalescence of ring carbons of carbohydrates' anomeric C1 placed at 102–105 ppm (de Aquino et al. 2019). The numerous resonances within the 110–140 ppm range correspond to the inclusion of unsubstituted and C‐substituted phenyl carbons, while those between 140 and 160 ppm centered at 151 ppm are related to O‐substituted carbons found in hydroxyl‐ and methoxy‐aromatic rings, typical of polyphenols and lignin derivatives (Verrillo et al. 2022). The last sharper carbon functionalities at 177 ppm refer to carbonyl bonds of acid groups in various dissolved structures, preceded by noticeable peaks of inorganic carbonates around 164–169 ppm. The molecular characteristics of CT samples were assessed through the dimensionless structural parameters, highlighting an overall shared distribution of hydrophobic and hydrophilic moieties in both CTs, with a greater content of apolar aromatic over aliphatic fractions in the CT‐BP sample, also indicated by the lower A/OA ratio (Table 1). In CT citrus, the presence of shoulders assigned to C4 carbons at 85–89 and the partial displacement of some C1 nuclei at 110 ppm (Figure S1) denoted the permanence of glycosidic linkages and the preservation of less decomposed polysaccharide and pectic components (de Aquino et al. 2019; Altieri et al. 2024). The tendentially small LigR values (Table 1) found in both CT samples confirmed the close correlation of O‐aryl‐C and methoxyl‐C spectral regions, thereby outlining a substantial dissolution of lignin fragments and phenolic elements in compost extracts (Verrillo, Cozzolino, et al. 2021; Verrillo, Salzano, et al. 2021).

#### 3.1.2 Off‐Line TMAH‐Pyr‐GC–MS

The thermochemolysis of CT samples released chiefly a large array of aryl molecules, originating from lignin constituents of compost extracts, thus confirming the outputs of NMR analyses (Verrillo, Cozzolino, et al. 2021; Verrillo, Salzano, et al. 2021). On the other hand, unlike NMR spectra, CT pyrograms revealed a relatively lower presence of carbohydrate (Table 2). This reduced detection was already observed in pyrograms of plant biomass, soil organic matter, and woody tissues. This is likely due to the limited efficiency of thermochemolysis in identifying saccharides within complex matrices (Verrillo et al. 2023; Altieri et al. 2024). The specific homologues of lignin units were associated with both abbreviations, corresponding to different lignin structural units (P for *p*‐hydroxyphenyl; Guaiacyl for 3‐methoxy, 4‐hydroxyphenyl; Syringyl for 3,5‐dimethoxy, 4‐hydroxyphenyl), and code numbers applied in dedicated literature to identify specific structural composition and modifications (Table 1). The survey of phenolic clusters in thermograms underlined the presence of both unaltered and microbially processed lignin materials. The latter included the aldehydic (G4, S4), ketonic (G5, S5), benzoic‐acid (G6, S6), and benzene‐propenoic (P18, G18) forms as main oxidised products of both di‐ and tri‐methoxy phenylpropane molecules (Table 1). The concomitant release of G10/11, G13, S10/11, and S13 (as either cis or trans isomers) may be related to the incorporation of partially decomposed lignin residues. Conversely, the identification of G14/G15 and S14/S15 enantiomers confirmed the persistence of unaltered lignified materials with intact intermolecular propyl ether linkages (de Aquino et al. 2019; Altieri et al. 2024). The lignin monomers issued from different decomposition stages may be used to evaluate the extent of depolymerization. In this respect, the ratio of peak areas of acidic structures over that of both the corresponding aldehydes (Ad/AlG = G6/G4, Ad/AlS = S6/S4) and over the sum of peak areas for the threo/erythro isomers (ΓG = G6/[G14 + G15];ΓS = S6/[S14 + S15]) is considered to act as a reliable indicator of the bio‐oxidative transformation of lignin‐cellulose fractions during composting processes (Verrillo, Cozzolino, et al. 2021; Verrillo, Salzano, et al. 2021). The highest values of these ratios were observed in the CT bell pepper, suggesting the larger release of oxidised, bioavailable low‐molecular‐weight lignin fragments into the compost extract. In contrast, the lower values observed for CT citrus suggest that less‐decomposed lignin structures remain associated with the lignocellulosic material (de Aquino et al. 2019) (Table 2).

**TABLE 2:** Structural index of lignin fractions in CT samples.

%	Ad/AlG	Ad/AlS	ΓG	ΓS
CT‐BP	6.0	8.8	3.8	3.9
CT‐C	2.4	2.7	1.1	1.7

*Note:* Ad/AlG = (G6/G4), Ad/AlS = (S6/S4); ΓG = G6/(G14 + G15), ΓS = G6/(S14 + S15).

### 3.2 Antioxidant Power

**FIGURE 1:**
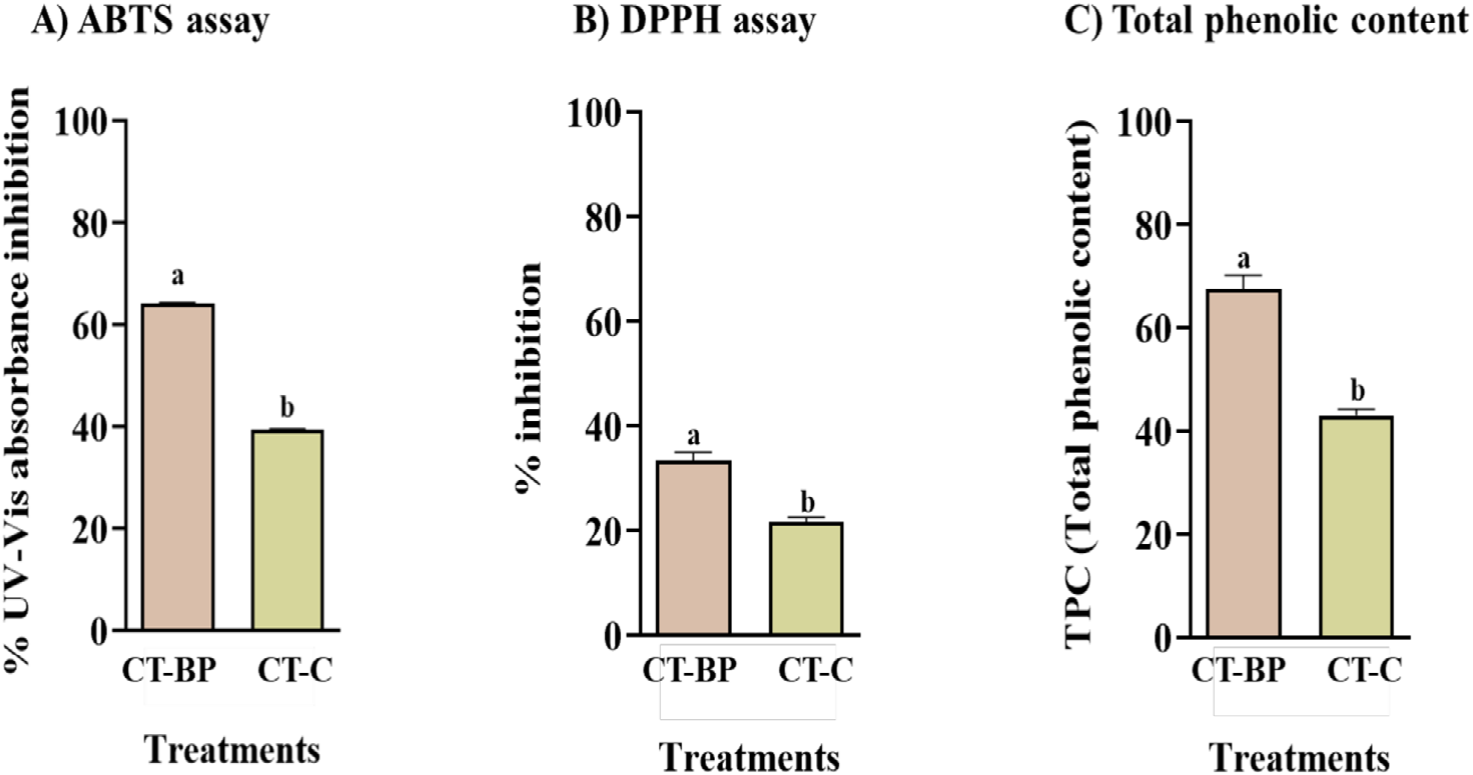
Antioxidant activity and total phenolic content of CT‐BP and CT‐C. Different letters denote statistically significant differences of each treatment compared to the control samples by the one‐way ANOVA test (*p* ≤ 0.05).

Organic matter is characterised by a strong correlation between the content of phenolic and quinonic moieties and their antioxidant and scavenging activities, primarily due to their behaviour as electron donors or acceptors, which influences their redox state (Klein et al. 2021). Since phenolic compounds are commonly associated with antioxidant activity (Alongi et al. 2025), the total phenolic content (TPC) of CT samples was also determined in this study. The results of the ABTS assay (Figure 1a) showed that UV–Vis absorbance inhibition was highest in compost teas derived from bell pepper (64.1%), whereas CT citrus exhibited a significantly lower inhibition value (39.3). A similar trend was observed in the DPPH assay, where CT bell pepper confirmed the greatest scavenging potential, followed in decreasing order by CT citrus (Figure 1b). The total phenolic content of CT samples followed the same trend as their antioxidant activity, with CT bell pepper exhibiting the highest TPC, followed by CT citrus (Figure 1c).

Although the Folin–Ciocalteu reagent is not highly specific for polyphenols, our findings align with previous studies that suggest a strong correlation between TPC and antioxidant activity in organic materials (Verrillo, Cozzolino, et al. 2021; Verrillo, Salzano, et al. 2021). Chemical composition analysis revealed that the antioxidant properties of CTs may be directly linked to their aromatic components, particularly lignin‐derived fragments. The observed correlation between antioxidant activity and TPC suggests that partially degraded lignin structures contribute significantly to the antioxidant capacity of compost teas (Li et al. 2024). Notably, nuclear magnetic resonance (NMR) and thermochemolysis analyses (Tables 1 and 2, Figure S2) confirmed that CT bell pepper contained higher amounts of oxidised lignin fragments compared to CT citrus. These findings are consistent with previous studies on phenolic materials, which have demonstrated that mono‐ and oligo‐hydroxylated benzene units exhibit substantial antioxidant properties (Verrillo et al. 2023). The antioxidant activity observed for CT bell pepper may be attributed to the degree of lignin fragmentation compared to CT citrus. Indeed, the lower values of degradation indexes found for CT citrus (Table 2) and the permanence of less decomposed cellulose inputs (Figure S1) suggest the presence of less bioavailable lignin structures still associated with lignocellulosic fragments. The antioxidant potential of natural organic materials is a key factor in the development of innovative and sustainable antioxidant systems (Marino et al. 2022).

However, to the best of our knowledge, no studies have previously investigated the antioxidant properties of compost teas derived from composted agricultural biomasses. Unlike conventional plant extracts, compost teas represent complex mixtures where composting enhances the bioavailability of phenolic metabolites while diversifying the redox‐active molecules. Such complexity may explain the stable antioxidant activity we observed, in agreement with studies linking humic‐derived phenolics to long‐term antioxidant potential (Klein et al. 2021). The results of this study indicate that compost teas represent a potential source of bioactive compounds with antioxidant properties, particularly when derived from lignin‐ and polyphenol‐rich feedstocks, such as bell pepper residues. These findings may open up new perspectives for the application of compost teas as functional agents for mitigating oxidative stress.

### 3.3 Antimicrobial Efficacy

Given the growing global concern regarding antimicrobial resistance and the urgent need for alternative strategies to combat pathogenic bacteria, exploring the antibacterial potential of natural bioactive compounds is strongly recommended. Bacterial infections pose a direct threat to human health due to the development of progressive resistance to drugs and the emergence of novel pathogenic strains (Vitiello et al. 2024). Natural products represent a specific source of bioactive metabolites, which can be exploited for their therapeutic effects (Messinese et al. 2024). In this context, the investigation of the antimicrobial properties of compost derivatives can be applied in medicine as a sustainable alternative to the use of industrial synthetic products for controlling human diseases (Castro‐Díaz et al. 2025). In this work, CTs were tested against some Gram‐positive ( *S. aureus* and *E. faecalis* ) and Gram‐negative bacterial strains (*E. coli and P. aeruginosa*) (Table 3), while albumin serum bovine–BSA was used as a reference negative control. An improved antimicrobial activity was found against Gram‐positive bacterial strains in both CT samples. The best performance against *S. aureus* was observed for CT‐BP, followed by CT‐C, with inhibition zones equal to 6.7 and 5.4 mm, respectively (Table 3). A similar trend was observed for the minimum inhibitory concentration assay (MIC), which revealed that all CTs were more effective than control against *S. aureus* and *E. faecalis* . Conversely, a lower susceptibility was found for Gram‐negative bacterial strains, such as *E. coli* and *P. aeruginosa* , which generally showed larger MIC values (Table 4). CT‐BP had the best antibacterial performance, with a low MIC value against *S. aureus* and *E. faecalis* . Conversely, a higher MIC value has been observed in the case of *E. coli* and *P. aeruginosa* when CT‐BP was tested (Table 4). The organic products employed here have revealed effective antimicrobial activity, which may be associated with the abundance of aromatic and phenolic fractions, as found in CT‐BP. The lower efficacy of CT‐C may be correlated with the lower content of bioavailable lignin molecules still integrated in lignocellulose oligomers, as indicated by both NMR spectra and thermochemolysis analysis. There is a link between the phenolic content of natural extracts and their antimicrobial properties against various human pathogens (Lima et al. 2019). In detail, previous studies have shown that lignin‐derived molecules and phenolic compounds act as redox mediators, contributing to antioxidant capacity while also exerting antimicrobial effects through membrane disruption and interaction with microbial proteins (Morena et al. 2022; Ali et al. 2024; Sholahuddin et al. 2024). Despite the growing understanding of these interactions, the precise mechanisms through which phenolic compounds exert antibacterial effects remain unclear. One prevailing theory suggests that phenolic compounds may disrupt bacterial cells by interacting with enzymatically active sites, leading to alterations in cell membrane permeability and compromising cell wall integrity, ultimately causing bacterial death (Chen et al. 2024). Other research has shown that polyphenols were more effective against gram‐positive bacteria than gram‐negative cells. This disparity is likely due to the structural differences in their cell walls and the presence of complex outer membranes in gram‐negative bacteria, which can serve as a barrier to prevent the penetration of many antimicrobial agents, including phenolic compounds (Donadio et al. 2021; Chen et al. 2023).

**TABLE 3:** Inhibition zones (mm) of CT‐BP and CT‐C by disk diffusion method (DDK). A mixture of antibacterial molecules (ampicillin and clavulanic acid) was employed as a control and tested at the same concentration as the compost teas (2 mg/mL). The coefficients of variation were invariably smaller than 6%.

DDK (mm)	*S. aureus*	*E. faecalis*	*E. coli*	*P. aeruginosa*
AMP + clavulanic acid	3.8 ± 0.04^a^	2.9 ± 0.1^a^	4.1 ± 0.08^b^	3.6 ± 0.05^a^
CT‐BP	6.7 ± 0.02^c^	6.3 ± 0.09^c^	5.4 ± 0.05^b^	4.1 ± 0.01^b^
CT‐C	5.4 ± 0.06^b^	5.3 ± 0.04^b^	4.7 ± 0.2^b^	3.6 ± 0.5^a^

*Note:* Different letters denote statistically significant differences of each treatment compared to the control samples according to the LSD test (*p* ≤ 0.05).

**TABLE 4:** Antibacterial activity against gram‐positive and gram‐negative bacterial strains of CT‐BP and CT‐C as determined by MIC (minimal inhibitory concentration). A mixture of antibacterial molecules (ampicillin and clavulanic acid) was employed as a control.

MIC (μg mL^−1^)	*S. aureus*	*E. faecalis*	*E. coli*	*P. aeruginosa*
AMP + clavulanic acid	2.1 ± 0.02^b^	1.9 ± 0.09^b^	3.4 ± 0.4^c^	3.8 ± 0.06^c^
CT‐BP	1.2 ± 0.04^a^	1.6 ± 0.06^b^	2.4 ± 0.05^b^	2.8 ± 0.02^b^
CT‐C	1.9 ± 0.02^b^	1.8 ± 0.07^b^	2.9 ± 0.2^b^	3.2 ± 0.06^b^

*Note:* Different letters denote statistically significant differences of each treatment compared to the control samples according to the LSD test (*p* ≤ 0.05).

Interestingly, the MIC values observed in our study for CT‐BP were markedly lower than those reported for direct *Capsicum* extracts. For example, Koffi‐Nevry et al. reported MIC values of 0.5 mg/mL for methanolic and aqueous extracts of *C. annuum* and *C. frutescens* against *S. aureus* (Koffi‐Nevry et al. 2012). Additionally, pure capsaicin and dihydrocapsaicin have been reported to inhibit bacterial growth with MICs ranging from 0.6–10 μg mL^−1^ depending on the bacterial species (Nascimento et al. 2014). Similarly, our CT‐BP displayed MICs of only 1.2 μg/mL against *S. aureus* and 1.6 μg/mL against *E. faecalis* , while maintaining activity in the low micromolar range against Gram‐negative bacteria such as *E. coli* (2.4 μg/mL) and *P. aeruginosa* (2.8 μg/mL). These results demonstrate that compost tea can actually equal or surpass the potency of direct extracts. A plausible explanation is that the composting process transforms native capsaicinoids and phenolics into more bioavailable derivatives or generates novel microbial metabolites with enhanced antimicrobial activity. Consistent with these findings, our study revealed that CT‐BP exhibited the strongest antibacterial activity attributed to the presence of high concentrations of polyphenols, suggesting a direct correlation between the molecular composition of compost tea and its antibacterial activity, further emphasising the importance of understanding the chemical nature of organic substances when evaluating their potential as antimicrobial agents.

### 3.4 Antiviral Activity

#### 3.4.1 CTs Inhibit Enveloped Virus Infection

Viral infections represent one of the leading causes of disease worldwide due to their complexity and diversity, which makes it very difficult to combat their spread without affecting the host cell homeostasis. The increase in migration, global travel, and urbanisation has made virus outbreaks a crucial concern for public health, especially when vaccines and antiviral therapies are still not available (Denaro et al. 2020). These aspects underscore the need to discover and identify new antiviral agents, which, in addition to traditional medicine, should primarily possess adequate selectivity, efficacy, and low toxicity (Akram et al. 2018). In recent years, bioactive compounds from organic waste have garnered significant interest in the scientific community (Iacono et al. 2024).

Viruses are characterised by a genome (either RNA or DNA) surrounded by a protein and/or lipid‐containing envelope (Cohen 2016). Since enveloped viruses enter host cells through their surface glycoproteins, we analysed whether CTs could impair their interaction with the host cell surface, preventing viral attachment and penetration. We assessed the antiviral potential against two enveloped viruses from different families. The first one, HSV‐1, is a linear double‐stranded DNA virus from the *Herpesviridae* family. HSV‐1 is one of the most prevalent and highly infectious viruses: more than 80% of people worldwide are affected by HSV‐1; among these, 40% exhibit at least one recurrent infection. The available treatments are based on several selective drugs, such as acyclovir, famciclovir, and trifluridine, that have led to unpleasant secondary effects, including the insurgence of drug‐resistant strains (Denaro et al. 2020).

**FIGURE 2:**
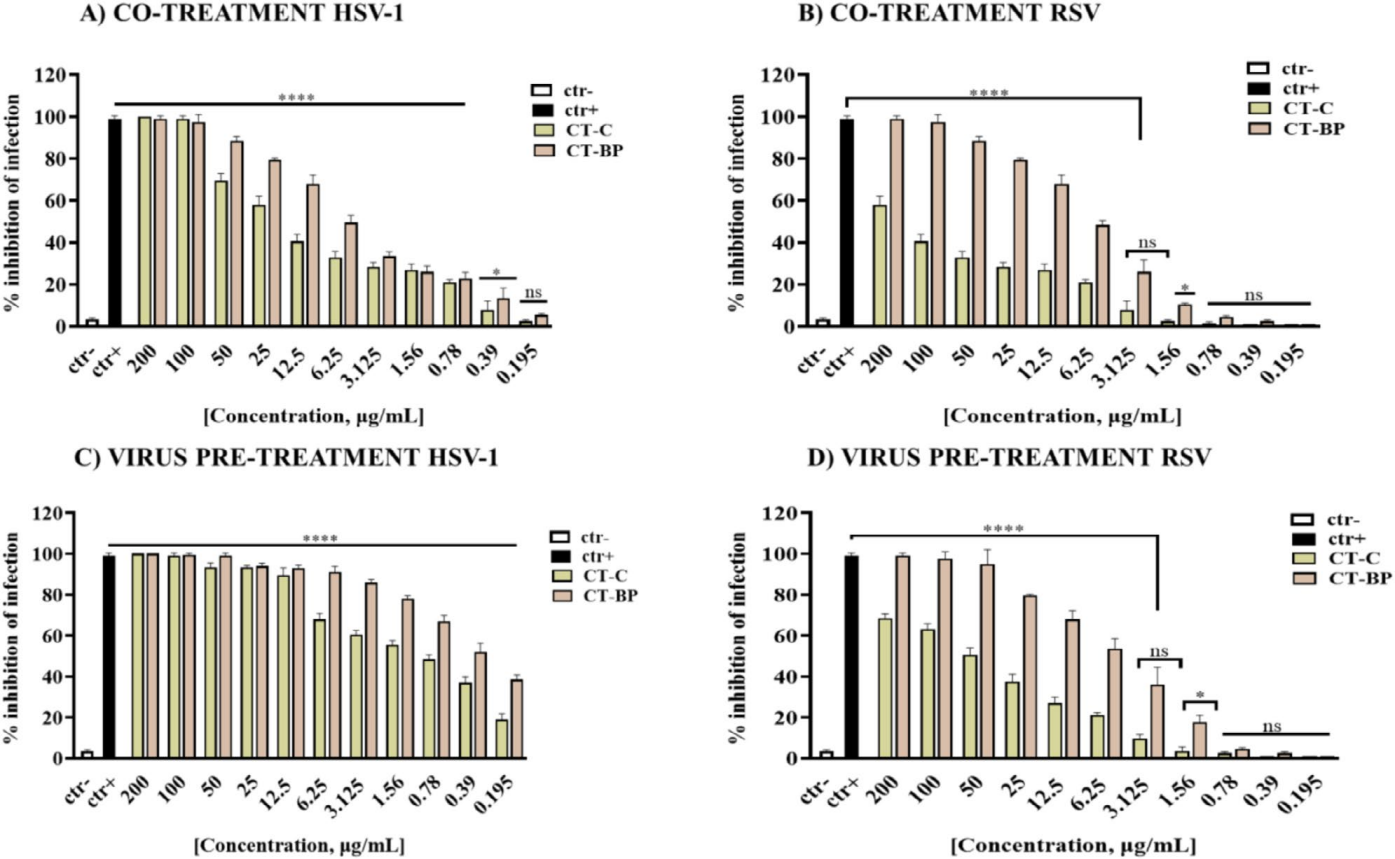
Antiviral activity of CTs against enveloped viruses. Two assays are reported here. (A) Co‐treatment assay against HSV‐1 and RSV in (B); (C) virus pre‐treatment assay against HSV‐1 and RSV in (D). For HSV‐1, rhamnolipid M15RL (50 μg/mL) was used in the co‐treatment and virus pre‐treatment (Giugliano et al. 2021); for RSV, Hylin‐a1 (50 μM) was used in co‐treatment and virus pre‐treatment assays (Chianese et al. 2023). Untreated but infected cells were used as controls (ctr−). The data represent the mean ± standard deviation (SD) of three independent experiments. ****p‐value < 0.0001; *p‐value < 0.03; ns: not significant.

To verify whether the inhibitory effect was correlated to the viral genotype, the second enveloped virus we chose was an RNA virus, that is, RSV. It belongs to the *Paramyxoviridae* family, whose members carry a negative single‐stranded non‐segmented RNA genome surrounded by a helical capsid (Gonnin et al. 2023) and an outer lipid bilayer. Respiratory viruses, including RSV, heavily contribute to the global burden of virus‐inflicted morbidity and mortality, and thus, the urgency for effective prevention and treatment options remains in high demand (Pollard and Bijker 2021). RSV infections mainly constitute a risk for infants and the elderly. Unfortunately, no effective vaccine is available, although many candidates are being developed (Pollard and Bijker 2021). The potential CT antiviral activity against HSV‐1 and RSV was evaluated on Vero‐76 cells using CT samples in non‐cytotoxic concentrations ranging from 0.195 to 200 μg/mL. In the co‐treatment assay, where cells were co‐treated with CT and viruses, CTs were able to inhibit infectivity in a dose‐dependent manner. Additionally, CT‐C showed an IC_50_ of 19.3 and 181 μg/mL against HSV‐1 and RSV, respectively. Meanwhile, CT‐BP inhibited viral infections more potently, with an IC_50_ very similar between the two viruses: 7.78 μg/mL against HSV‐1 and 6.8 μg/mL against RSV (Figure 2a,b). To understand at which stage of viral infection CTs acted, we investigated whether CTs directly impacted viral particles or indirectly interacted with the pre‐infected (cell pre‐treatment assay) or post‐infected host cells (post‐treatment assay). We observed a substantial improvement in antiviral activity in virus pre‐treatment, in which CT‐C acted with an IC_50_ of 1.21 and 52.7 μg/mL against HSV‐1 and RSV, respectively; on the other hand, CT‐BP was still active at very low concentrations against both viruses (IC_50_ values of 0.38 and 5.63 μg/mL for HSV‐1 and RSV, respectively) (Figure 2c,d).

On the other hand, CTs did not show any activity in either cell pre‐treatment or post‐treatment experimental conditions, implying that they had no effect during viral replication or cell‐to‐cell spread (Figure S3).

These results indicated that CTs interfered during the extracellular infection phase, targeting the viral envelope. According to other studies, vegetable by‐products have been tested against different viral families and, in most cases, have shown virucidal activity when in direct contact with the virus (Iacono et al. 2024). In these cases, extracts from by‐products, particularly rich in polyphenols, damage the viral envelope by hindering the attachment and adsorption phases and preventing infection. For example, a recent study reports the B‐type procyanidin condensed tannins, polymers of (−)‐epicatechin, from the leaves of *Mitragyna speciosa* (kratom) as potent virucidal agents against SARS‐CoV‐2. Through activity‐guided fractionation and structural analyses, researchers identified high–molecular weight tannins responsible for the antiviral effect, while the monomer (−)‐epicatechin itself showed no activity. The tannin fraction displayed strong in vitro virucidal activity with an EC₅₀ of about 8.38 μg/mL and a high selectivity index, suggesting potential as a natural antiviral lead (Sureram et al. 2024).

The specific molecular interactions that affect viral infectivity are still unclear. The most common hypothesis is that the hydroxyl groups of polyphenols interact with positively charged domains of the envelope glycoproteins (Gescher et al. 2011). To the best of our knowledge, a few antiviral data from citrus and bell pepper natural sources are present against RSV (Xu et al. 2014, 2015), but their anti‐herpetic application has been successfully explored.

For instance, a previous study has shown that methanolic extracts of *Capsicum annuum* possess antiviral activity against HSV‐1 and HSV‐2, attributed to the ability of the extract's phytochemical compounds to block viral penetration and modify viral and cellular receptors (Hafiz et al. 2017; Romero‐Luna et al. 2022). Recently, the anti‐HSV‐1 potential of *Citrus aurantium* leaf extracts has been exploited for the same purpose (Mejri et al. 2024). This mode of action is consistent with previous observations that plant‐derived polyphenols, particularly hydroxylated compounds, can disrupt the interaction between viral glycoproteins and host cell receptors, thereby blocking viral entry (Coskun et al. 2025).

Interestingly, our CT‐BP exhibited stronger inhibition than some direct extracts, suggesting that composting may enhance the release of active compounds or generate novel bioactive derivatives. CT‐BP exhibited higher antiviral activity than CT‐C towards both viral types, with higher capsaicinoid and polyphenol content, indicating promise as a therapeutic agent for combating respiratory diseases.

#### 3.4.2 CT Did Not Affect Naked Virus Infection

To validate that the CTs' target was the viral envelope, we tested them against PV‐1, a non‐enveloped virus belonging to the *Picornaviridae* family. PV‐1 has a positive‐strand RNA genome encapsulated only by an icosahedral protein coat. PV is the causative agent of poliomyelitis, which mainly affects children under 5 years of age, and is a target of global eradication by the World Health Organization (WHO) (Arita and Fuchino 2023).

**FIGURE 3:**
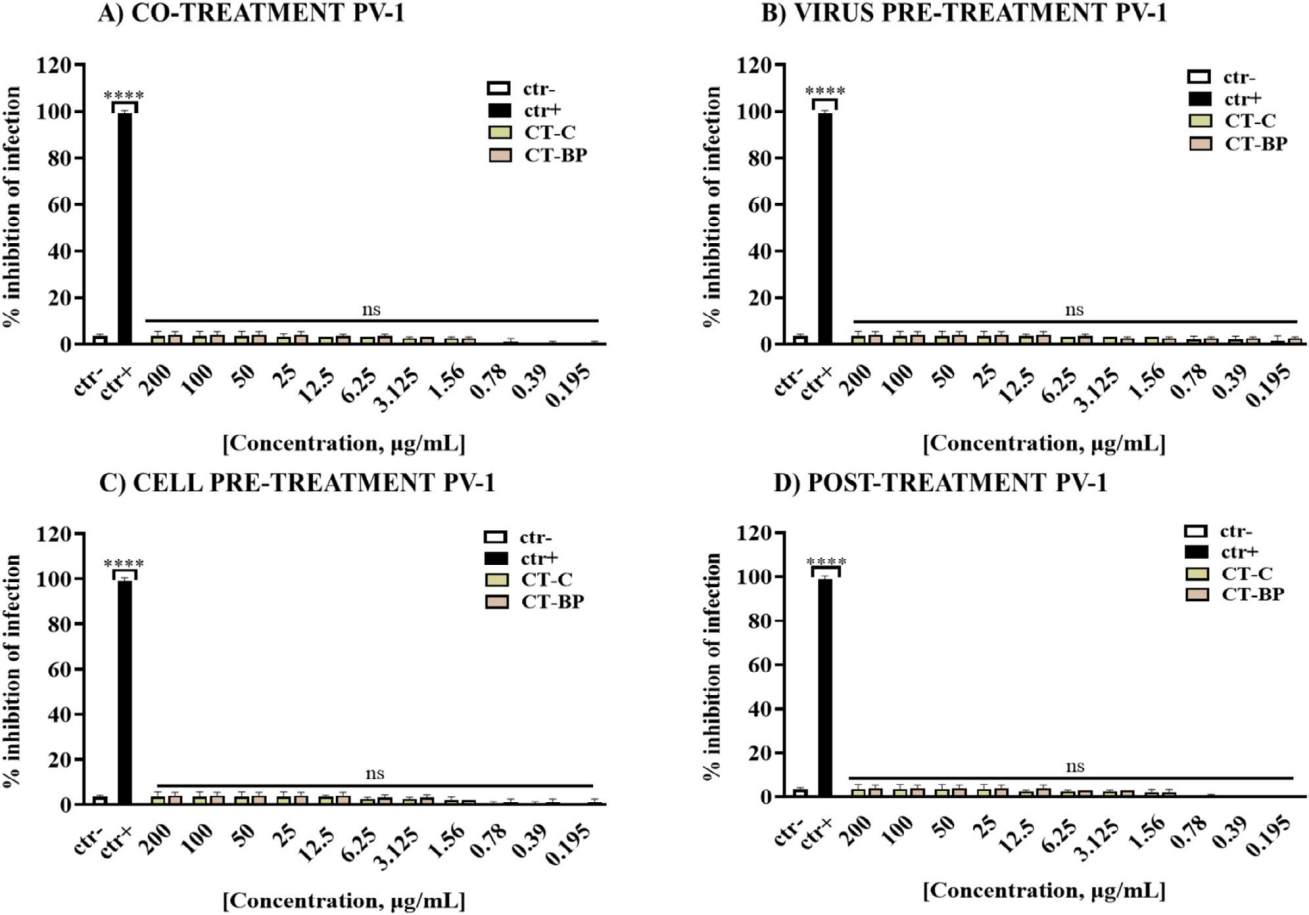
Antiviral activity of CTs against PV‐1, a naked virus. Four assays are reported here. (A) Co‐treatment assay; (B) virus pre‐treatment assay; (C) cell pre‐treatment assay; (D) post‐treatment assay. For PV‐1, pleconaril (2 μg/mL) was used in co‐treatment and virus pre‐treatment, and WIN51711 (5 μg/mL) and protein 2C (10 μM) in cell pre‐treatment and post‐treatment, respectively (Ambrosino et al. 2023); finally, untreated but infected cells were used as controls (ctr−). The data represent the mean ± standard deviation (SD) of three independent experiments. ****p‐value < 0.0001; ns: not significant.

Antiviral activity against PV‐1 was tested in the same experimental conditions used for HSV‐1 and RSV. However, no relevant viral inhibition was recorded in the tested concentration range of 0.195–200 μg/mL, confirming that CTs could act directly on the viral envelope (Figure 3).

In our study, CTs emerged as natural ingredients and sources of bioactive compounds with antibacterial and antiviral activities, illustrating a direct and sustainable use of agricultural waste. Capsaicinoids and phenolic compounds are among the bioactive compounds found in CTs, and their antimicrobial effects primarily result from cell membrane disruption and pore formation in pathogenic bacteria. Studies on viruses are scarce, yet it seems they can interfere with the binding of envelope glycoproteins to host cell receptors during the absorption phase (Hafiz et al. 2017, Romero‐Luna et al. 2022).

A holistic and strategic approach is essential for unlocking their full potential in various applications, particularly for safeguarding public health.

## 4 Conclusion

This study demonstrates that compost teas derived from agro‐industrial wastes possess significant antimicrobial and antiviral properties, particularly those from bell pepper residues (CT‐BP), which showed higher polyphenol and oxidised lignin content. CT‐BP was more effective against gram‐positive bacteria and enveloped viruses (HSV‐1 and RSV), whereas both teas showed limited activity against gram‐negative bacteria and no effect on non‐enveloped viruses. These findings support compost teas as sustainable, natural bioactive agents and align with circular economic principles.

## 5 Study Limitations

CT efficacy is attributed generically to polyphenols and lignin derivatives, but we are currently working to identify the individual compounds responsible for the inhibitory activity. Future research should focus on optimising extraction processes and refining bioactive formulations. Moreover, the antiviral and antimicrobial activities were evaluated on cell cultures and laboratory bacterial strains. Confirmation in vivo or on more complex models will be explored for translating the results into clinical applications. Our data suggest a direct effect on the bacterial membrane and the viral envelope, but further studies will be conducted to reveal the CT's exact mechanism of action.

## Author Contributions

**Carla Zannella:** resources, funding acquisition, writing – review and editing, supervision. **Annalisa Chianese:** software, visualization, data curation. **Anna De Filippis:** funding acquisition, resources, supervision, writing – review and editing. **Riccardo Spaccini:** writing – review and editing, supervision. **Roberta Della Marca:** investigation, methodology, writing – original draft, visualization.

## Funding

This work was supported by the POST DOCTORAL FELLOWSHIP 2024, Fondazione Veronesi.

## Conflicts of Interest

The authors declare no conflicts of interest.

## Supporting information


**Figure S1:** NMR spectroscopy (^13^C CPMAS NMR) of CT‐BP and CT‐C samples.
**Figure S2:** Thermochemolysis pyrograms of CT‐BP and CT‐C samples.
**Figure S3:** Antiviral activity of CTs against enveloped viruses. Two assays are reported here. (A) Cell pre‐treatment assay against HSV‐1 and RSV in (B); (C) Post‐treatment assay against HSV‐1 and RSV in (D). Untreated but infected cells were used as controls (ctr−).

## Data Availability

The data that support the findings of this study are available on request from the corresponding author. The data are not publicly available due to privacy or ethical restrictions.
