# Supplementary material for: Compost Tea as a Natural Bioactive Solution: Unlocking the Antimicrobial and Antiviral Potential of Bell Pepper and Citrus Wastes
Source: Environ Microbiol Rep. 2026 Jan 27;18(1):e70260. doi: 10.1111/1758-2229.70260 (PMC12841596; doi:10.1111/1758-2229.70260)
Supplement: Supplementary file 1 — Figure S1: NMR spectroscopy (13C CPMAS NMR) of CT‐BP and CT‐C samples. Figure S2: Thermochemolysis pyrograms of CT‐BP and CT‐C samples. Figure S3: Antiviral activity of CTs against enveloped viruses. Two assays are reported here. (A) Cell pre‐treatment assay against HSV‐1 and RSV in (B); (C) Post‐treatment assay against HSV‐1 and RSV in (D). Untreated but infected cells were used as controls (ctr−). [file EMI4-18-e70260-s001.docx]

**Compost Tea as a Natural Bioactive Solution: Unlocking the Antiviral and Antimicrobial Potential of Bell Pepper and Citrus Wastes**

Verrillo M.^1,2^, Della Marca R.^3^, Cozzolino V. ^1,2^, Chianese A.^3^, Zannella C.^3^, Galdiero M.^3,4^, Spaccini R.^1,2^, De Filippis A.^3^

1. Department of Agricultural Sciences, University of Naples Federico II, 80055 Portici, Naples, Italy.

2. Interdepartmental Research Centre on Nuclear Magnetic Resonance (NMR) for the Environment, Agro-food and New Materials (CERMANU), University of Naples Federico II, 80055 Portici, Naples, Italy.

3. Department of Experimental Medicine, University of Campania "L.Vanvitelli", 80138 Naples, Italy.

4. Complex Operative Unit of Virology and Microbiology, University Hospital of Campania "Luigi Vanvitelli", 80138 Naples, Italy.

**Supplementary Materials**

**
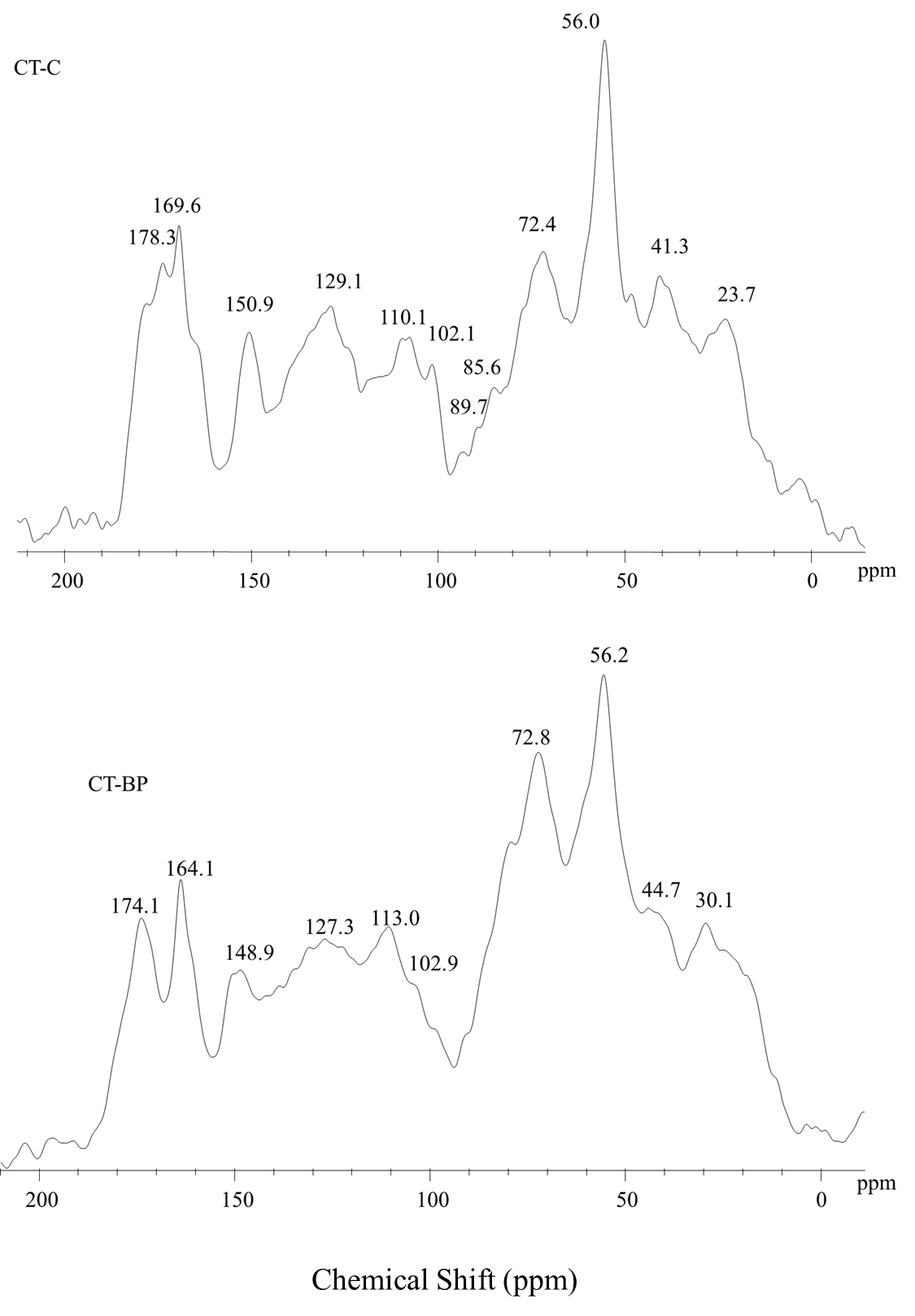
**

**Fig. S1. NMR spectroscopy (^13^ C CPMAS NMR) of CT-BP and CT-C samples.**


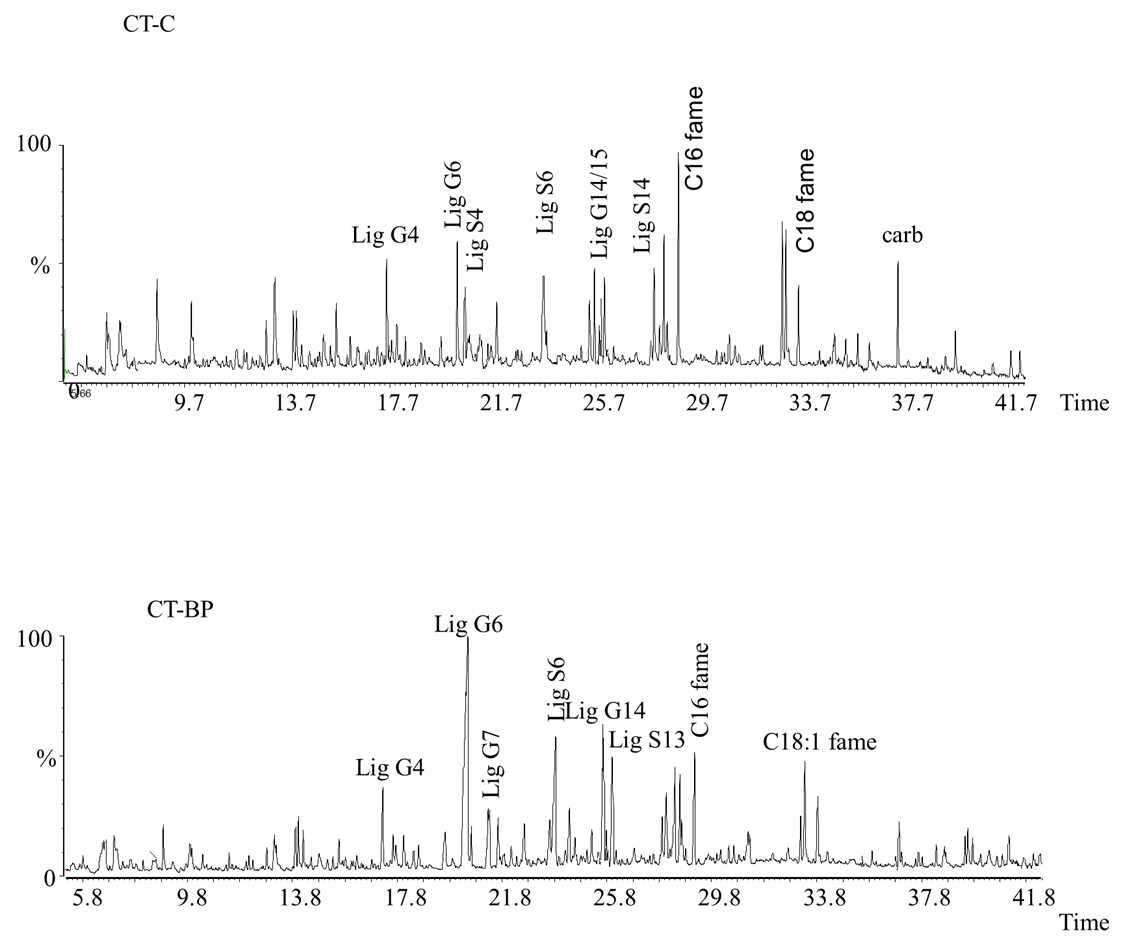


**Fig. S2. Thermochemolysis pyrograms of CT-BP and CT-C samples.**


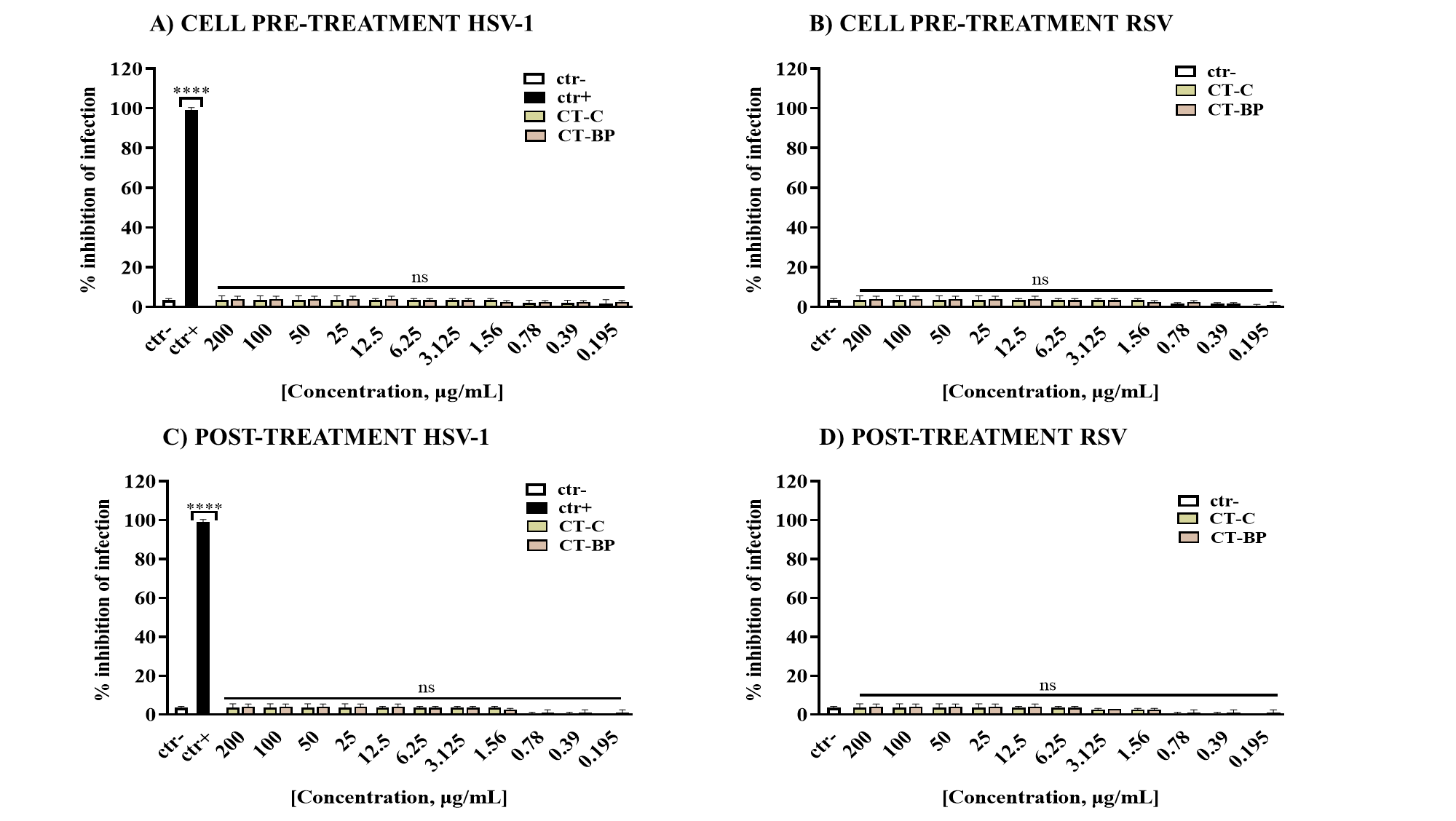


**Fig. S3**. Antiviral activity of CTs against enveloped viruses. Two assays are reported here. (**A**) Cell pre-treatment assay against HSV-1 and RSV in (**B**); (**C**) Post-treatment assay against HSV-1 and RSV in (**D**). Untreated but infected cells were used as controls (ctr -).

For HSV-1, dextran-sulfate (1 µM) in cell pre-treatment, and acyclovir (5 µM) in post-treatment (Chianese, et al., 2023). The data represent the mean ± standard deviation (SD) of three independent experiments. ****: p-value < 0.0001; ns: not significant.
